# Cytokinin-Regulated Sucrose Metabolism in Stay-Green Wheat Phenotype

**DOI:** 10.1371/journal.pone.0161351

**Published:** 2016-08-31

**Authors:** Wenqiang Wang, Qunqun Hao, Fengxia Tian, Qinxue Li, Wei Wang

**Affiliations:** 1 State Key Laboratory of Crop Biology, Shandong Key Laboratory of Crop Biology, College of Life Sciences, Shandong Agricultural University, Tai’an, Shandong, 271018, China; 2 College of Life Science and Technology, Nanyang Normal University, Nanyang, Henan, 473061, China; Murdoch University, AUSTRALIA

## Abstract

A wheat stay-green mutant, *tasg1*, was observed to exhibit significantly delayed senescence in the late developmental stage. The photosynthetic capacity of the flag leaf was greater in *tasg1* than in wild type (WT) plants. In addition, the grain volume of *tasg1* was significantly higher than that of WT at the early filling stage. The content of various cytokinins (CKs) in the grain was significantly higher in *tasg1* than in WT and was accompanied by an upregulated expression of some cell cycle-related genes. Examination of the metabolism of soluble sugars in *tasg1* and WT revealed that the concentrations of glucose (Glu), fructose (Fru), and sucrose (Suc) were higher in the flag leaves and grains of *tasg1* than in WT plants. The activities of sucrose-phosphate synthase (SPS), sucrose synthase (SuSy), and cell wall invertase (CW-invertase) were higher in *tasg1*, suggesting an altered metabolism and transport of soluble sugars. Furthermore, when *tasg1* was treated with the CK inhibitor lovastatin, the activity of invertase was inhibited and was associated with premature senescence phenotype. However, the activity of invertase was partially recovered in *tasg1* when treated with 6-benzylaminopurine (BAP). The trend of change in the concentrations of Glu, Fru, and Suc was similar to that of invertase. Our results suggest that CKs might regulate the stay-green phenotype of *tasg1* by regulating the invertase activity involved in Suc remobilization.

## Introduction

Senescence is an internally programmed degenerative process leading to death in plants. Premature leaf senescence could be responsible for low grain yield. Compared to wild type (WT) plants, stay-green or non-yellowing mutants of various plant species have been reported to maintain leaf greenness for longer time during senescence and are ideal materials for studying the mechanisms underlying plant senescence. Some stay-green mutants maintain photosynthetic activity for longer durations and are, therefore, expected to have a higher yield [1, 2]. However, the mechanisms underlying the stay-green or delayed senescence phenotype remain unclear, to date.

Cytokinins (CKs) are known to play important roles in plant growth and developmental processes, including senescence [3]. These processes are linked to the demand for carbohydrates, regulation of assimilate partitioning [4], sink strength [5], and source-sink relationships [6].

Sink strength is affected by cell division. CKs play important roles in the regulation of different cell cycle phases, including the G1/S transition, progression through S phase, and G2/M transition [7, 8]. In addition, CKs are involved in the production of carbohydrates. In previous studies, CKs were demonstrated to promote chloroplast biogenesis, increase the photosynthetic rate, and affect the abundance of proteins associated with photosynthesis, including Rubisco [9] and chlorophyll a/b-binding protein of the light-harvesting complex [10]. CKs affect the distribution of nutrients and further modulate sink strength as indicated by their ability to establish local metabolic sinks, which has been demonstrated by mobilisation of radiolabeled nutrients, such as sugars, from other parts of the plant to CK-treated areas [5].

Sucrose (Suc) metabolism and transport are very important for growth and senescence. These processes depend on sucrose-phosphate synthase (SPS), sucrose synthase (SuSy), and invertase (C-Invertase, V-Invertase, CW-Invertase) activities. SPS and SuSy play important roles in regulating the synthesis of sucrose [11, 12]. The activity of invertase has been reported to be dominant during initiation and expansion of sink [13].

Plants contain neutral invertases, localized to the cytosol (C-Invertase), and acidic invertases, localized to the vacuoles (V-Invertase) and cell wall (CW-Invertase) in the apoplast [14]. In particular, extracellular invertase (CW-Invertase) has crucial functions, both in source-sink regulation and in supplying carbohydrates to sink tissues, and is, therefore, a central modulator of sink activity [15, 16, 17]. CKs are also involved in the regulation of invertase activity; extracellular invertase activity is usually high in tissues with an elevated cytokinin concentration [18].

A wheat stay-green mutant, *tasg1*, was previously generated via mutation breeding of HeSheng2 (HS2) cultivar in our laboratory [19]. We observed that *tasg1* was a functional stay-green mutant with delayed senescence. The content of CKs, the stability of proteins in thylakoid membranes, and the antioxidant capacity of *tasg1* were consistently higher than those of WT during senescence [20, 21]. This resulted in a higher biomass of *tasg1* compared to that of WT under drought stress. In the present study, we found that the grain volume in *tasg1* was higher than that in WT at the early filling stages. We, therefore, made an attempt to (i) analyze the involvement of cell division and CKs in the changed grain volume in *tasg1*; (ii) investigate the Suc metabolism in the leaves (source) and grains (sink) of *tasg1* and analyze its involvement in the high biomass and stay-green phenotype of *tasg1*, and (iii) determine the relationship between CK and Suc metabolism in the stay-green phenotype of tasg1. These data are expected to contribute to a better understanding of the CK metabolism and delayed senescence in wheat.

## Materials and Methods

### Plant materials

A wheat stay-green mutant, *tasg1*, was generated via mutagen breeding in our laboratory, using the mutagen EMS (ethyl methane sulfonate) applied to HeSheng 2 (HS2), a common wheat cultivar (*Triticum aestivum* L.).

### Field experiments

Field experiments were carried out at the farm of Shandong Agricultural University, China. Six interspersed plots (four m^2^) were selected randomly in the field (random block design); the experiment was conducted in triplicate with three plots being used for each genotype. Wheat seeds were sown in eight lines separated by a width of 25 cm, at a depth of two cm, and with an interval of five cm between two seeds in the same line. The growth and development of the wheat plants were managed according to conventional agricultural techniques.

### Laboratory experiments

The methods for obtaining the wheat seedlings were modified according to Tian et al. [21]. WT and *tasg1* seeds were germinated on filter paper moistened with water for 24 h at 25 ± 1°C after being sterilized with 0.2% sodium hypochlorite. The seeds (hydroponic) were then placed in a well-ordered fashion on a nylon gauze sheet at the appropriate density and cultured in trays (25 cm × 18 cm × 5 cm) containing water. These trays were placed in a growth chamber at 25 ± 1°C under a 12 h light (300 μmol m^–2^ s^–1^) dark cycle and a relative humidity of 70%.

To induce leaf senescence in wheat seedlings, we used smaller than two-week-old seedlings in a hydroponic system without nutrients. *Tasg1* showed significantly delayed senescence phenotype in the late growth stage (nine days) compared with WT.

For lovastatin treatment, the wheat seedlings were cultivated in a 40 μM lovastatin solution (containing 1% acetone) when they were grown to 7 d.

For 6-benzylaminopurine (BAP) treatment, the wheat seedlings were sprayed in a 100 μM BAP solution (Sigma) with 0.02% (v/v) Tween-20.

### Determinations of photosynthetic parameters in the field

Flag leaves were analysed for photosynthetic gas exchange parameters, including Pn, transpiration rate (E), stomatal conductance (Gs) and intercellular CO_2_ concentration (Ci); the method was modified according to Hui et al. [19].

### Chlorophyll a fluorescence analysis

The actual PSII efficiency under irradiance (ΦPSII) and the maximal photochemical efficiency of PSII (Fv/Fm) were measured according to Tian et al. [21].

### Determination of CK content

The methods for the extraction and purification of isopentenyl adenine (IPA), zeatin riboside (ZR), and dihydrozeatin riboside (DHZR) were modified according to Degenhardt et al. [22]. The fresh leaves or roots (0.5g) were extracted and purified by passing C18-Sep-Pak cartridges. The mouse monoclonal antigens and antibodies against DHZR, ZR, and IPA used in ELISA were produced at the Phytohormones Research Institute (China Agricultural University). ELISA was performed on a 96-well microtitration plate. Each well on the plate was coated with 50μl sample and 50μl antigens (0.25 μg mL^-1)^ against the hormones. The coated plates were incubated for 0.5h at 37°C in a wet box. After washing four times with PBS + Tween 20 (0.1% [v/v]) buffer (pH 7.4), each well was filled with 100μl antibodies (20 μg mL^-1^) and incubated for 0.5h at 37°C in a wet box. The plate was rinsed four times with above buffer and 100μl color-appearing solution containing 2 mg mL^-1^ OPD and 0.008% (v/v) H_2_O_2_was added to each well. The reaction progress was stopped by adding of 50μl 2M H_2_SO_4_ per well when the 2,000 ng mL ^-1^standard had a pale color. Color development in each well was detected using an ELISA Reader at optical density A_490._

### Determination of soluble sugars and enzyme activities

Soluble sugars (Glu, Fru, and Suc) were extracted and measured photometrically using a coupled enzymatic assay as described previously [23]. The measurement of enzyme activities was based on published assays for SPS [24] and SuSy [25].

To assay activities of cell wall, vacuolar and cytosolic invertases, the extraction was carried out as described by Roitsch et al. [26]. The homogenization buffer was 50 mM Hepes-KOH, pH 7.5, 1 mM EDTA, 3 mM MgCl_2_, 0.1 mM PMSF, 2% (v/v) glycerol and 1 mM benzamidine. Thehomogenate was mixed for 30 min at 4°C before centrifugation. The invertase reactions were carried out in K-phosphate/citrate buffer, pH 6.8 for cytosolic invertae, and pH 4.5 for vacuolar and cell wall invertases, with 125 mM sucrose as a substrate. The reaction was incubated for 1 h at 26°C and stopped by incubating for 5 min at 95°C. The amount of glucose liberated in the reaction was determined as above. Protein concentrations were determined according to GENMED Bradford (GENMED, China).

### Quantitative reverse transcription PCR (qRT-PCR) analysis

Total RNA from wheat flag leaves and grains, which were grown under normal conditions during the whole filling stage, was isolated according to the manufacturer’s protocol (flag leaf, Trizol Up, Trans, China; grain, RNAprep Pure Plant Kit, TianGen, China). qRT-PCR was carried out in Quantiative analysis was performed using the Bio Rad CFX Manager system. This method normalizes the expression of a specific gene versus a control reference with the formula 2-ΔΔCT. In this study, the mRNA levels for two stably expressed genes, tubulin and actin, were evaluated as control genes for qRT-PCR analyses. The information from all of the genes in the qRT-PCR experiments are listed in Table 1http://www.sciencedirect.com/science/article/pii/S0885576515000338 - tbl1.

**Table 1:** qRT-PCR primers used in the present study.

Gene Name	Sequence (5′ to 3′)
*Tubulin*	F: ATCTGTGCCTTGACCGTATCAGG; R: GACATCAACATTCAGGACACCATC
*Actin*	F: CGAAGCGACATACAATTCCATC; R: GAACCTCCACTGAGAACAACAT
*CycD2*	F: CTCCAGCAACATGCTACCAA; R: ACACACCTGAAGCACAGCAC
*His4*	F: ATCAACGACCACGAACCCTA; R: CTTCCTCGAGAACGTCATCC
*PCNA*	F: CGACATCATCACCATCAAGG; R: TGTCCATGAGCTTCATCTCG
*CDKB*	F: CGGGATTTAAAGCCACAAAA; R: CTCTGCTTAGCCCAAGATCG
*Sucrose-inducible*	F: TTTTGTCGGCCATAGAAACC; R: CTGCCATAAAAGTGGGCAAT
*Sucrose starvation-inducible*	F: TTCGTCTTGCTCGATACACG; R: CATACCGACCCATCAATTCC
*Ivr1*	F: CATGAGGGGGATCGCGGTGTTGTA; R: ACCCTTGACGGCCTTGTTGCTGAC
*Ivr3*	F: GTGGAGGATGGCAGTTGGTGGTGA; R: GCTCTATTCCTTGATGGCTGA

### Statistical analysis

All experiments and determinations were conducted at least in triplicate. The data processing system procedures (DPS, Shandong Agricultural University, China) were used to perform statistical analyses. All pairwise comparisons were analysed using Duncan’s test. Differences between the mean values obtained for wheat lines or treatments were compared using Duncan’s multiple range tests at 0.05 probability levels (*, *P* < 0.05; **, *P* < 0.01).

## Results

### Comparison of phenotypic-course dynamics of *tasg1* during filling stage and WT

**Fig 1:**
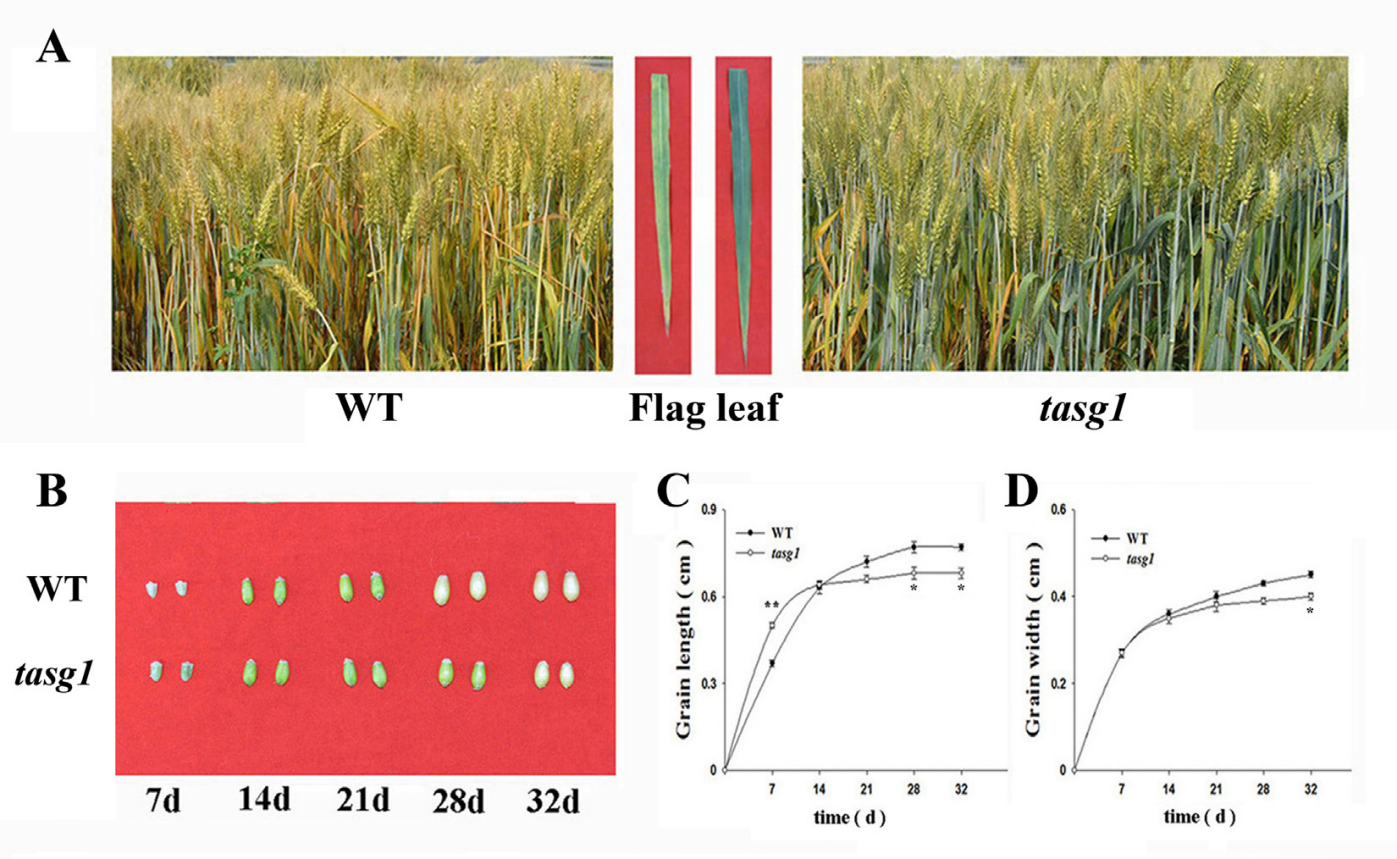
The different phenotypic-course dynamics of *tasg1* and WT during the filling stage in the field. (A) The stay-green phenotype of *tasg1* compared with WT at the late-filling stage (28 d after flowering); (B) The grain phenotypes of WT and *tasg1* at different days after flowering; (C-D) The dynamics of wheat grain parameters including Grain length (C) and Grain width (D). Error bars indicate means ± SE of data from thirty replicates. *, *P* < 0.05; **, *P* < 0.01.

In our previous study, *tasg1* was observed to delay senescence during the late developmental stage [19]. In the present study, it was found that, delayed senescence (Fig 1A) was accompanied by significantly higher grain volume in *tasg1* at the early filling stage (7 d) (Fig 1B). The grain length was longer in *tasg1* than in WT (Fig 1C), but there was no obvious difference in the grain width between *tasg1* and WT (Fig 1D). However, at the late filling stage (32 d), the grain volume of *tasg1* was lower than that of WT. Besides the length, the grain width in *tasg1* was also significantly lower than that in WT (Fig 1C and 1D).

### Changes in biomass and photosynthesis in *tasg1*

We quantified single stem biomass in the present study. The weight of stem in *tasg1* was significantly higher (by 14.1%) than that in WT at the late filling stage (S1A Fig), which was consistent with the greater lodging-resistance characteristic in *tasg1* at the late growth stage (data not shown). In addition, compared to WT, the weight and area of all the leaves on a single stem were higher in *tasg1* (S1B Fig); however, the number of leaves was not significantly different in the late growth stage [21]. These data demonstrate higher single stem biomass in *tasg1* than in WT.

**Fig 2:**
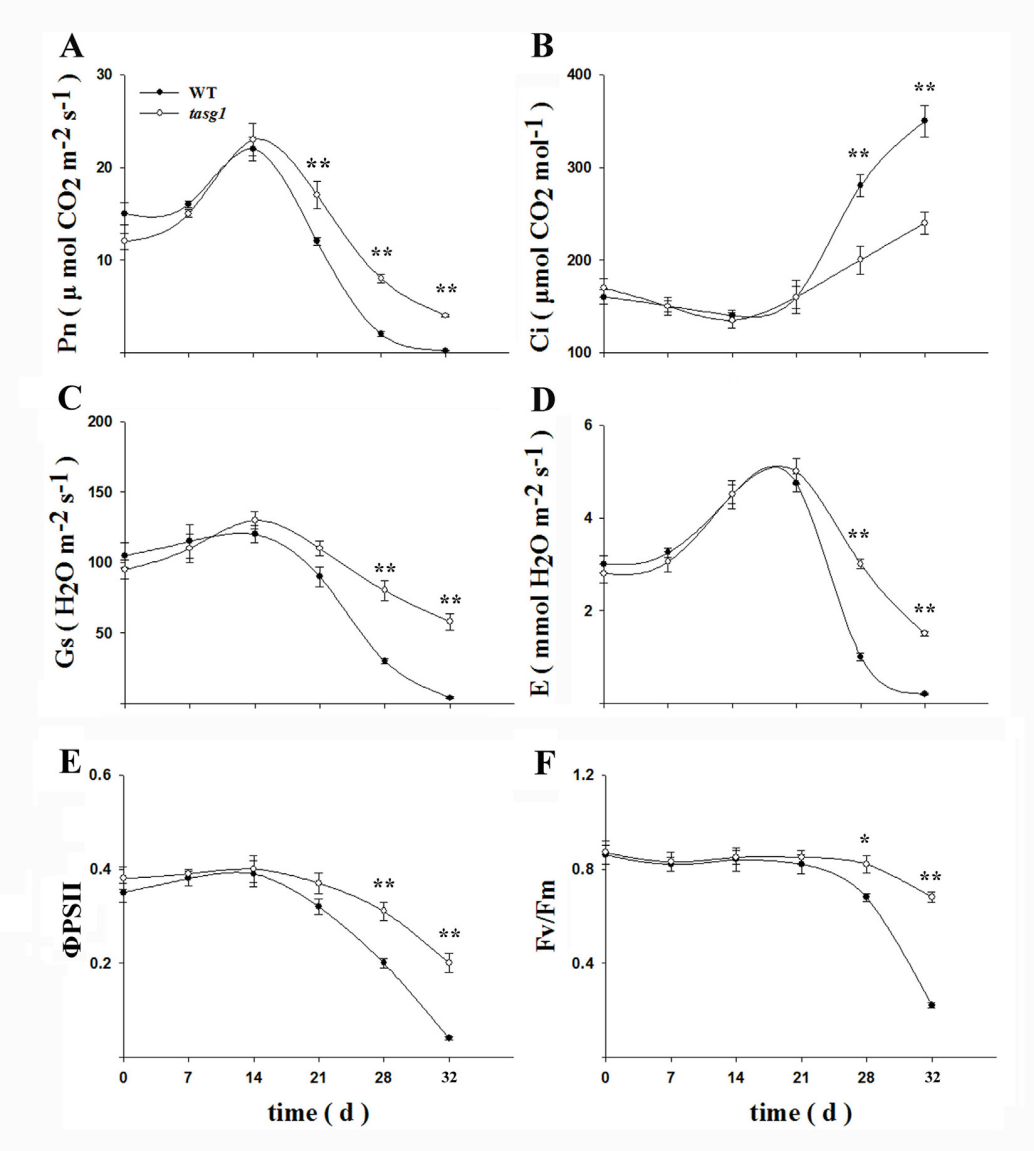
Changes in photosynthetic parameters. (A) Net photosynthetic rate (Pn), (B) Intercellular CO_2_ concentration (Ci), (C) Stomatal conductance (Gs), (D) Transpiration rate (E), (E) The actual PSII efficiency under irradiance (ΦPSII), and (F) Maximal photochemical efficiency of PSII (Fv/Fm) of flag leaves in WT and *tasg1* at the filling stage in the field. Error bars indicate mean ± SE of data from three replicates. *, *P* < 0.05; **, *P* < 0.01.

As photosynthesis is the major source of plant biomass, photosynthetic parameters were determined in *tasg1*. The difference in the change in photosynthetic rate (Pn) (Fig 2A) between *tasg1* and WT indicated the difference in light energy fixation during leaf growth. In particular, during the late developmental stage, the Pn of *tasg1* was significantly higher than that of WT. At the early stage (before 21 d), the transpiration rate (E) was not significantly different between *tasg1* and WT (Fig 2D), but at the late filling stage, it was significantly higher (275% more) in *tasg1* than in WT. The change in stomatal conductance (Gs) (Fig 2C) was consistent with the changes in E at the late filling stage. After 28 d of flowering, intercellular CO_2_ concentration (Ci) in *tasg1* (Fig 2B) was significantly lower than that in WT (44.2% lower), which was consistent with the change in Pn (Fig 2A).

The change in ΦPSII (Fig 2E) gradually decreased throughout the filling stage, but it was significantly higher in *tasg1* than in WT at the late growth stage. The trend of Fv/Fm (Fig 2F) was consistent with that of ΦPSII. The data in Fig 2 shows that the photosynthetic efficiency of the source (flag leaf) in *tasg1* was significantly higher than that in WT at the late filling stage; this might have been due to the major involvement of higher biomass of *tasg1* compared to that of WT (S1 Fig).

### Changes in CK content and cell cycle-related gene expression in *tasg1*

**Fig 3:**
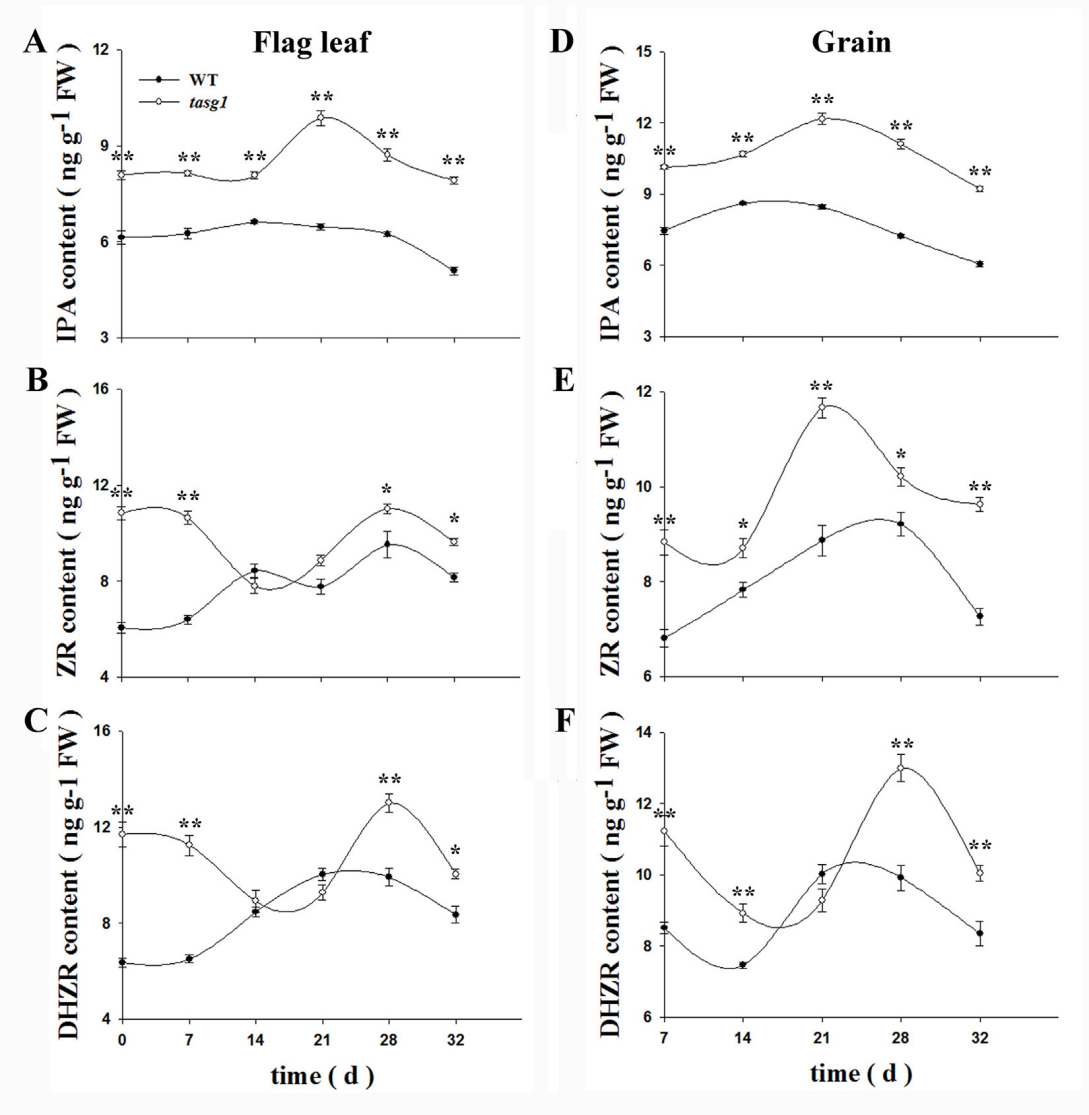
Changes in CK content in wheat grain and leaves of WT and *tasg1* at the filling stage in the field. (A, D) Contents of isopentenyl adenine (IPA), (B, E) zeatin riboside (ZR), and (C, F) dihydrozeatin riboside (DHZR) in the flag leaf and grain, respectively. Error bars indicate mean ± SE of data from three replicates. *, *P* < 0.05; **, *P* < 0.01.

We measured the contents of CKs (including IPA, ZR, and DHZR) in the flag leaves and grains of *tasg1* and WT at the filling stage. In flag leaves, the changes in ZR (Fig 3B) and DHZR (Fig 3C) showed a similar trend, that is, at the early (before 14 d) and late (after 28 d) stages, their concentrations in *tasg1* were significantly higher (about two-fold) than those in WT. During the entire filling period, the concentration of IPA was significantly higher than that in WT (Fig 3A). In grains, the change in concentration of CKs was similar to that in flag leaf during filling (Fig 3D–3F), but the total concentration of CKs was higher than that in the flag leaf.

**Fig 4:**
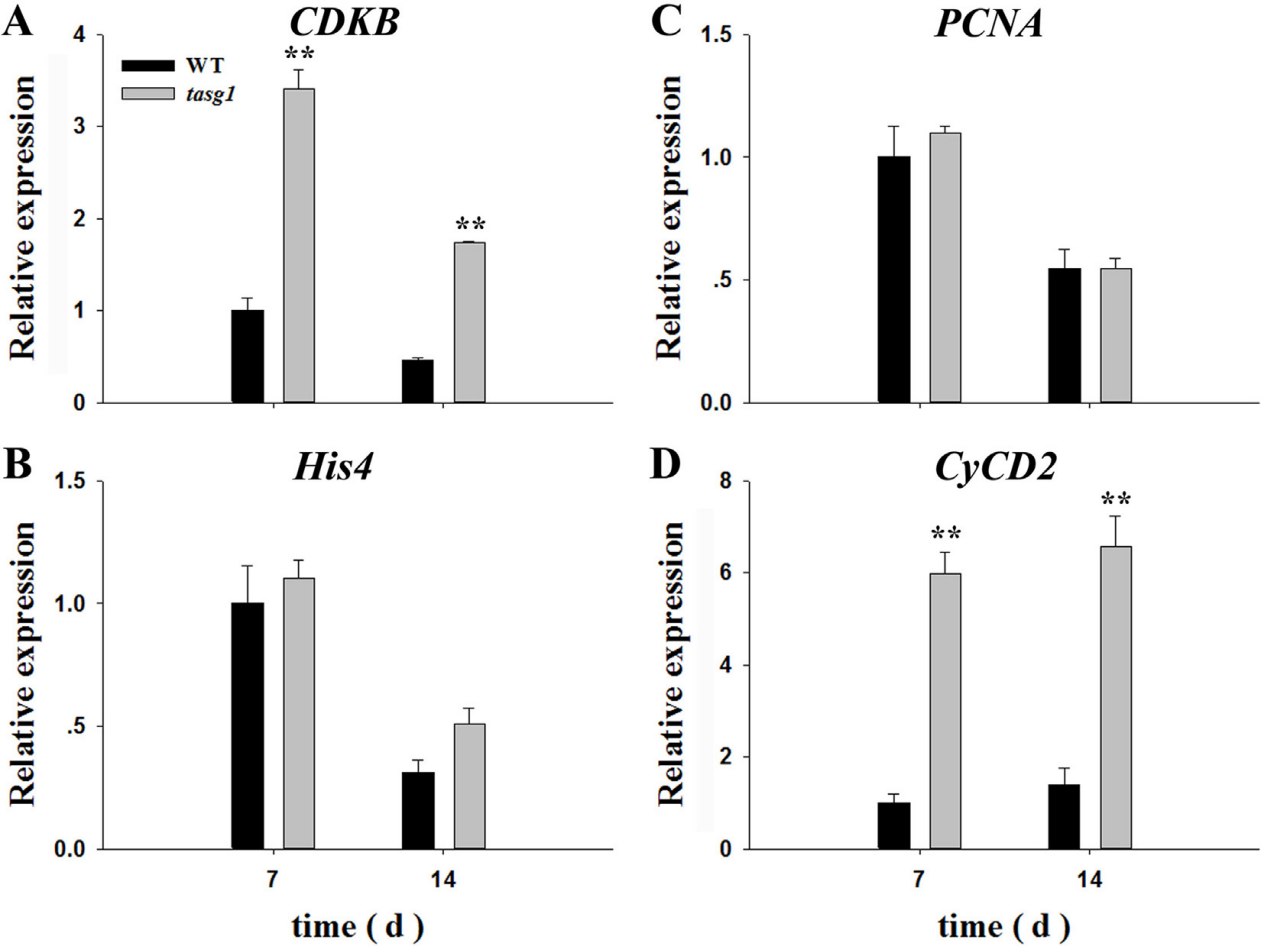
Relative expression of cell cycle-related genes in grains of WT and *tasg1* at the early filling stage (7 d, 14 d) in the field. (A) *CDKB*, (B) *His4*, (C) *PCNA*, and (D) *CycD2*. Error bars indicate mean ± SE of data from six replicates. *, *P* < 0.05; **, *P* < 0.01.

To understand the mechanism behind the bulk grain content (sink) in *tasg1* (Fig 1B) at the early filling stage (7 d, 14 d), we analyzed the expression of putative wheat orthologs of cell cycle genes during mitosis in WT and *tasg1*. Among the detected genes (Fig 4), the relative expression of *cyclin-dependent kinase B* (*CDKB*, G2/M phase) (Fig 4A) was significantly higher in *tasg1* than in WT at the early filling stage, with relative levels being 3.4- (7 d) and 3.7-fold (14 d) higher than the levels in WT. However, the expression of *histone H4* (*His4*, S phase) (Fig 4B) and *proliferating cell nuclear antigen* (*PCNA*, S phase) (Fig 4C) was downregulated, and no significant difference in expression was observed between WT and *tasg1*. The expression of *cyclin D2* (*CycD2*, G1 phase) was increased at the early filling stage (Fig 4D), and it was higher in *tasg1* (by 498% on 7 d and 371% on 14 d) than in WT. These results suggest that the expression of *CDKB* and *CycD2* might play an important role in increasing the grain volume (sink) in *tasg1* compared to WT at the early filling stage.

### Changes in Suc-metabolism in *tasg1*

**Fig 5:**
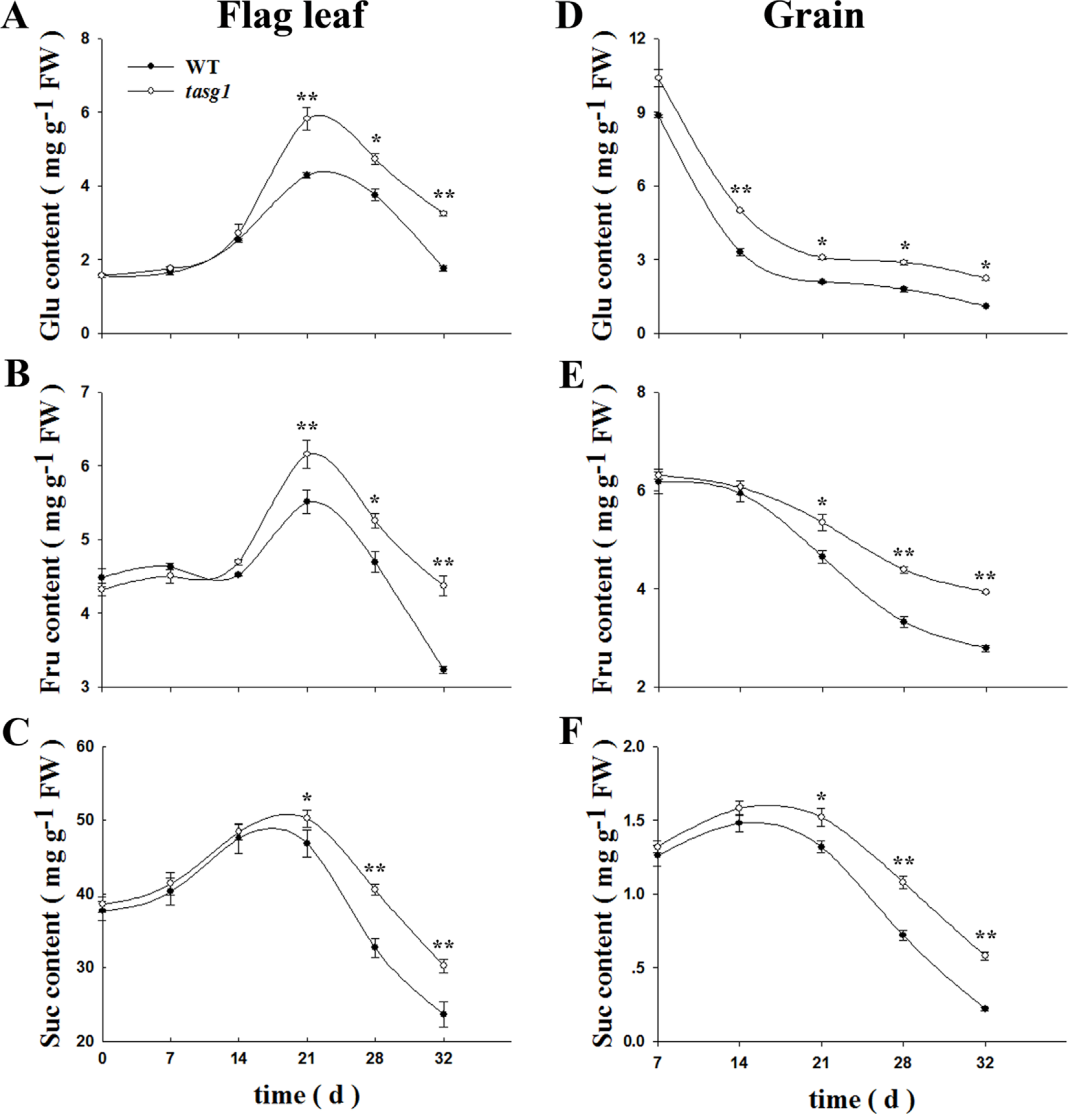
Content of carbohydrates in source (leaf) and sink (grain) tissues of WT and *tasg1* at the filling stage in the field. The measurements include, (A) Flag leaf glucose (Glu), (B) Flag leaf fructose (Flu), (C) Flag leaf sucrose (Suc), (D) Grain Glu, (E) Grain Flu, and (F) Grain Suc contents. Error bars indicate mean ± SE of data from three replicates. *, *P* < 0.05; **, *P* < 0.01.

The concentration of soluble sugars (including Glu, Flu, and Suc) was measured in flag leaf and grains in WT and *tasg1* (Fig 5). The contents of all the soluble sugars showed a gradual increase in the flag leaves at the early filling stage followed by a rapid decline at the late filling stage (Fig 5A–5C). However, the contents of all the sugars were significantly higher by 44.8 (Glu), 22.3 (Flu), and 26.8% (Suc) in *tasg1* than in WT at the late growth stage. In grains, the change in soluble sugar content was almost the same as that in the flag leaf (Fig 5D–5F). These results indicated that the production of soluble sugars in the flag leaf and their accumulation in grains were significantly higher in *tasg1*, signifying a potentially stronger ability for grain filling in *tasg1* than in WT.

**Fig 6:**
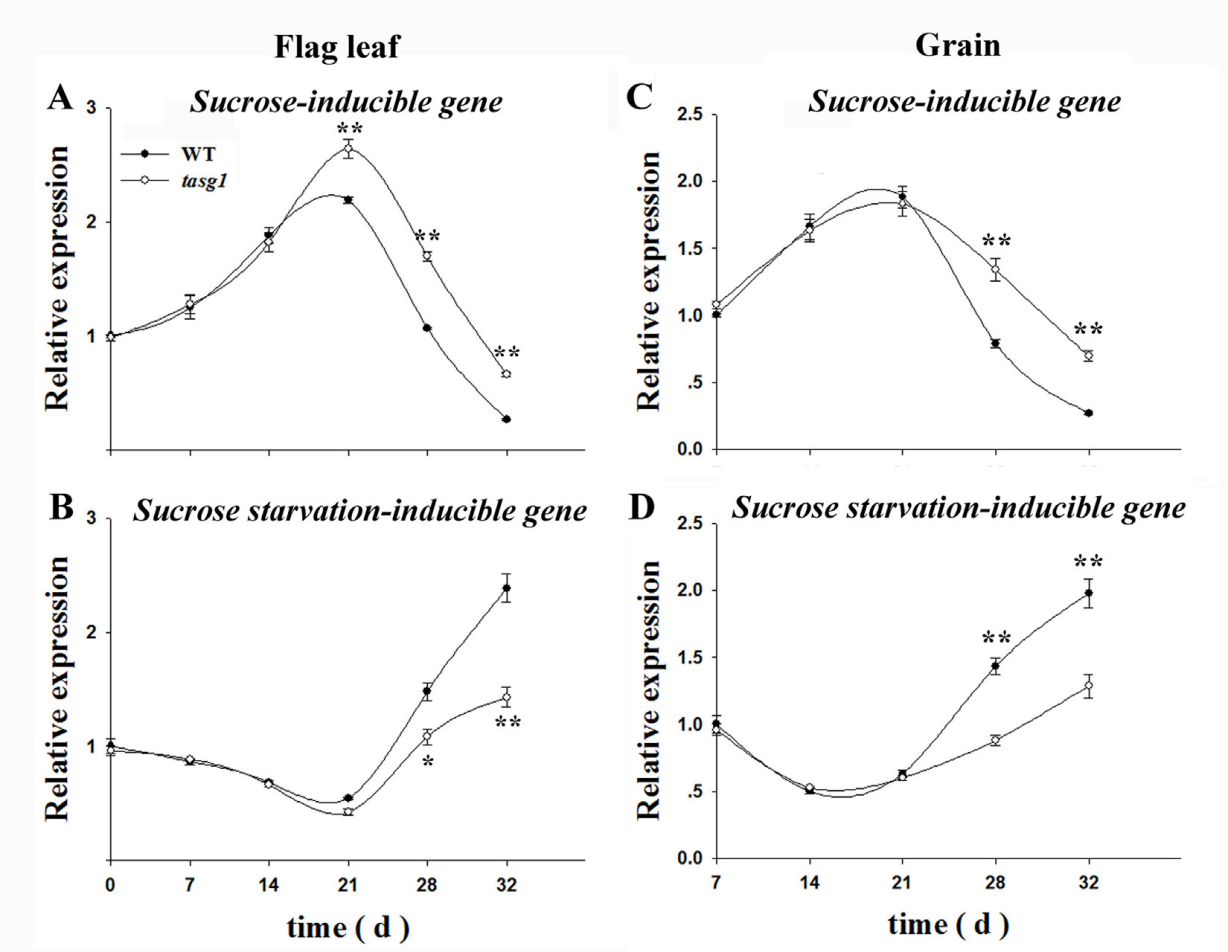
Relative expression of Suc-inducible and Suc-starvation inducible genes. (A) Suc-inducible gene expression in flag leaves, (B) Suc starvation-inducible gene expression in flag leaves, (C) Suc-inducible gene expression in grains, (D) Suc starvation-inducible gene expression in grains. Error bars indicate mean ± SE of data from six replicates. *, *P* < 0.05; **, *P* < 0.01.

Changes in Suc-related gene expression were examined in the flag leaf and grains at the filling stage, including the expression of wheat cDNAs AK332443 (*Suc-inducible gene*) and AK334107 (*Suc starvation-inducible gene*) [27], which was similar to *Arabidopsis* genes in response to Suc. The expression of the wheat *Suc-inducible gene* was upregulated in flag leaves of *tasg1* (Fig 6A). In contrast, the *Suc starvation-inducible gene* was expressed at a lower level in *tasg1* than in WT (Fig 6B). The expression pattern in grains (Fig 6C and 6D) was similar to that in flag leaf. These data suggest that the *Suc-related genes* may be involved in higher soluble sugar accumulation in *tasg1* than in WT during the late filling stage (Fig 5).

**Fig 7:**
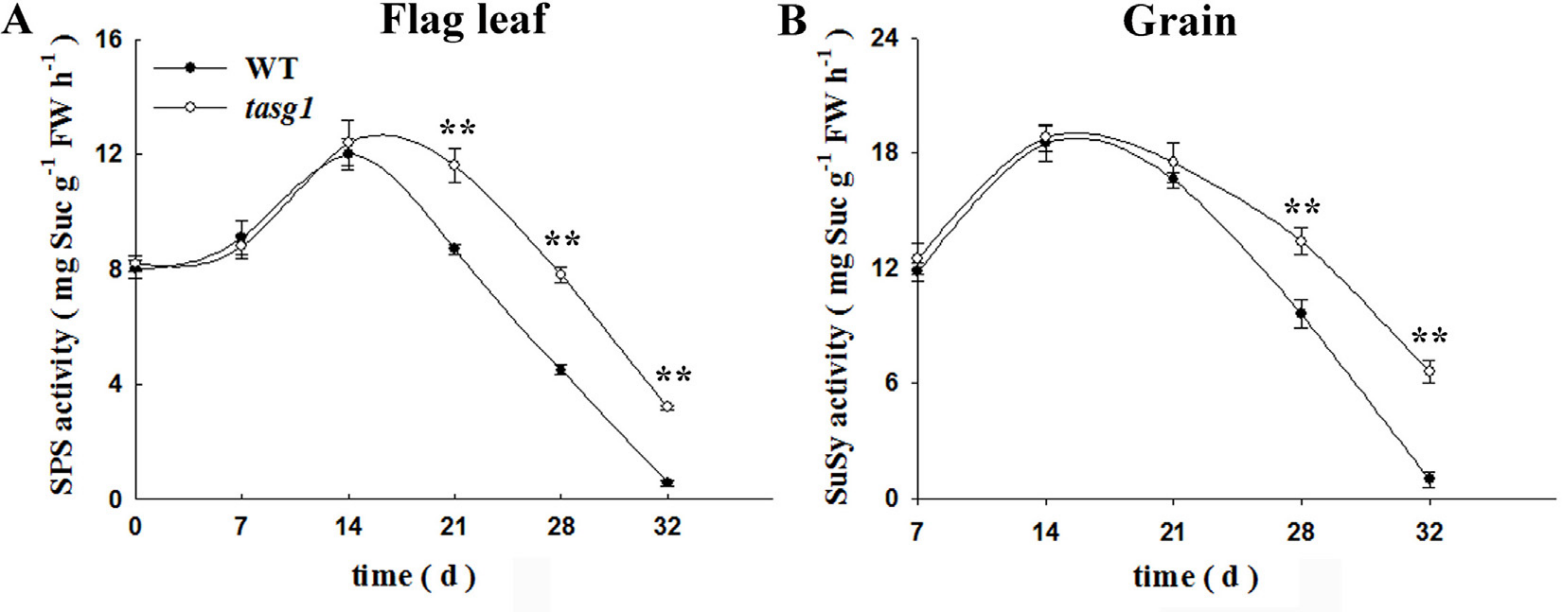
The activities of sucrose metabolism-related enzymes in source (leaf) and sink (grain) tissues of WT and *tasg1* at the filling stage in the field. (A) Sucrose phosphate synthase (SPS) activity, (B) Sucrose synthase (SuSy) activity. Error bars indicate mean ± SE of data from three replicates. *, *P* < 0.05; **, *P* < 0.01.

We subsequently detected the activity of enzymes related to Suc metabolism. The activities of SPS (flag leaf, Fig 7A) and SuSy (grain, Fig 7B) were significantly higher (by 121% and 73.6%, respectively) in *tasg1* than in WT at the late filling stage. The high activity of SPS might play an important role in the synthesis of Suc in the flag leaf of *tasg1*. Higher activity of SuSy in grains was observed to be favorable for the decomposition of Suc into Glu and Fru (Fig 5D and 5E).

**Fig 8:**
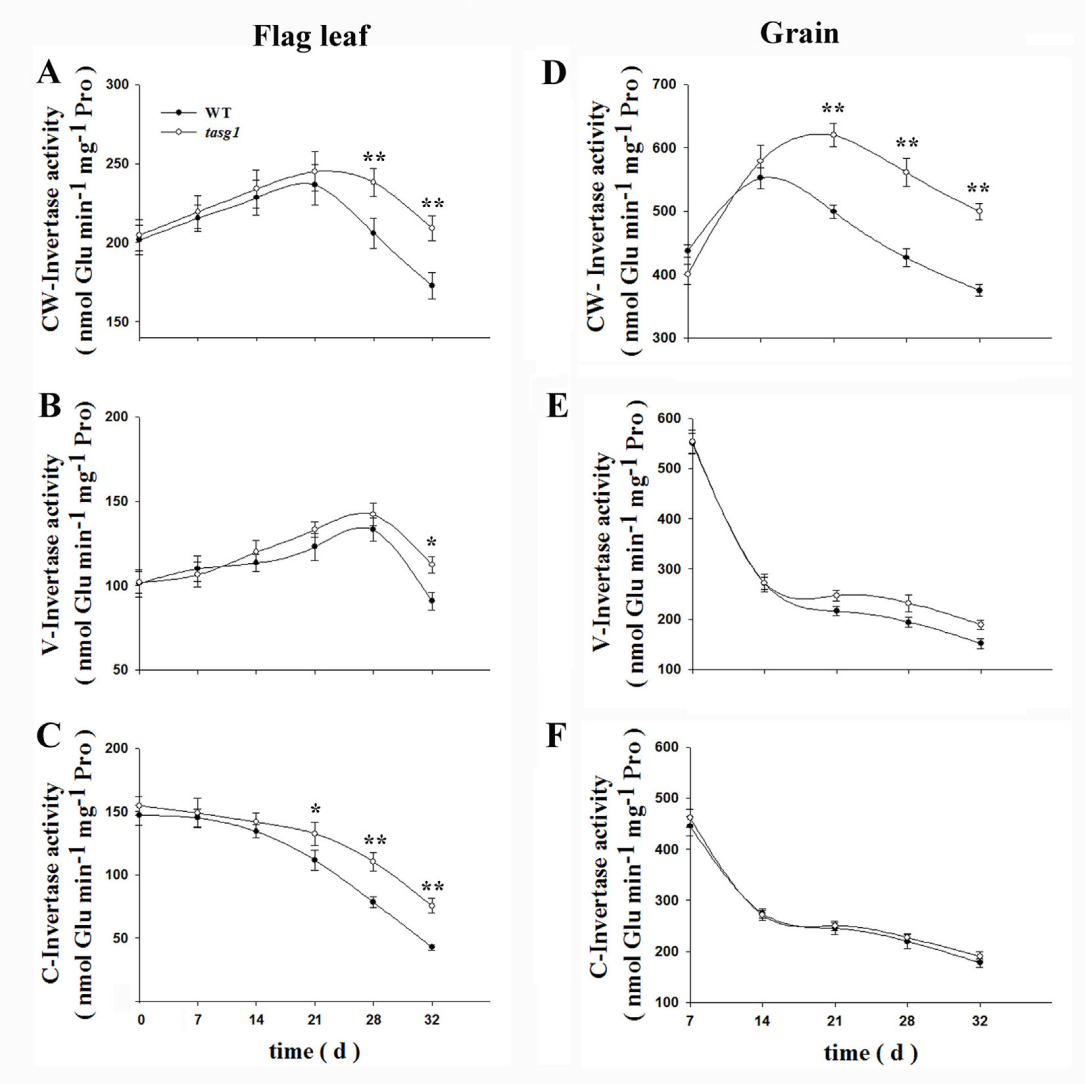
Invertase activities in source (leaf) and sink (grain) tissues of WT and *tasg1* at the filling stage in the field. (A) Flag leaf CW-Invertase, (B) Flag leaf V-Invertase, (C) Flag leaf C-Invertase, (D) Grain CW-Invertase, (E) Grain V-Invertase, (F) Grain C-Invertase. Error bars indicate mean ± SE of data from three replicates. *, *P* < 0.05; **, *P* < 0.01.

Furthermore, we measured the activities of enzymes related to Suc transport. The activities of CW-Invertase (Fig 8A), V-Invertase (Fig 8B), and C-Invertase (Fig 8C) in flag leaves were not different between WT and *tasg1* at the early filling stage. However, at the late filling stage, the invertase activity in *tasg1* was significantly higher than that in WT, especially for CW-Invertase and C-Invertase. In grains, the activity of CW-Invertase (Fig 8D) in *tasg1* was significantly higher (by 88.9%) than that in WT at the late filling stage, but no significant differences were observed in the activities of V-Invertase (Fig 8E) and C-Invertase (Fig 8F) throughout the filling stages. The data in Figs 6–8 together suggest that the metabolism and transport of Suc was more active in *tasg1* than in WT at the late filling stage.

**Fig 9:**
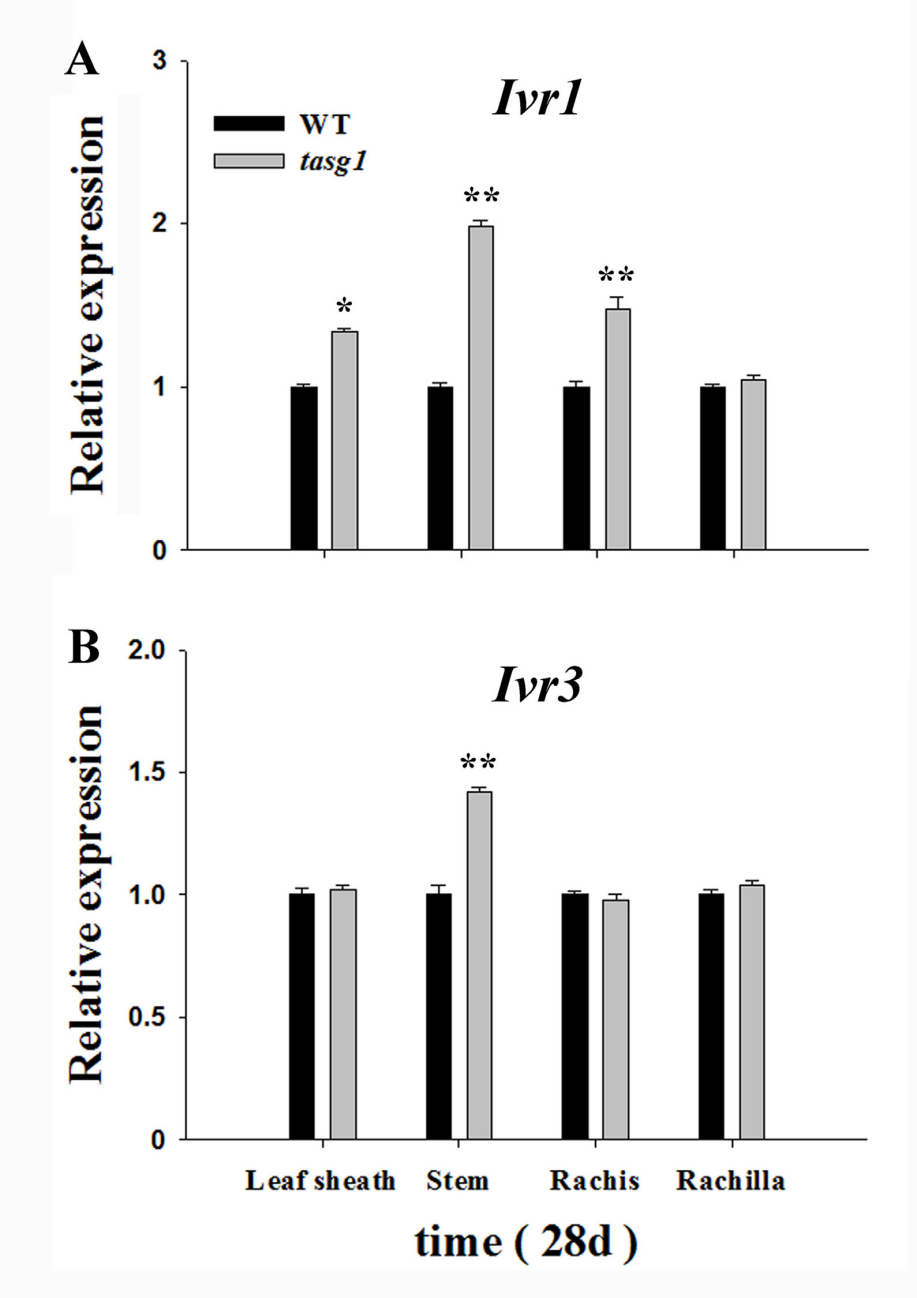
Relative expression of cell wall invertase-related genes in different tissues (Leaf sheath, Stem, Rachis, and Rachilla) of WT and *tasg1* at the late filling stage (28 d) in the field. (A) *Ivr1* expression, (B) *Ivr3* expression. Error bars indicate mean ± SE of data from six replicates. *, *P* < 0.05; **, *P* < 0.01.

*Ivr1* and *Ivr3* are the two CW-Invertase coding genes [28]. We assessed the relative expression of *Ivr1* (Fig 9A) and *Ivr3* (Fig 9B) in different tissues of WT and *tasg1* at the late filling stage (28 d). The results demonstrate that the expression of *Ivr1* was higher in leaf sheath (by 34.2%), stem (by 98.1%), and rachis (48.5%) of *tasg1* compared to that in the corresponding tissues of WT. However, the expression of *Ivr3* was significantly higher only in stems (by 42.3%) of *tasg1* compared to that in WT.

### Involvement of CK in the regulation of invertase activity

**Fig 10:**
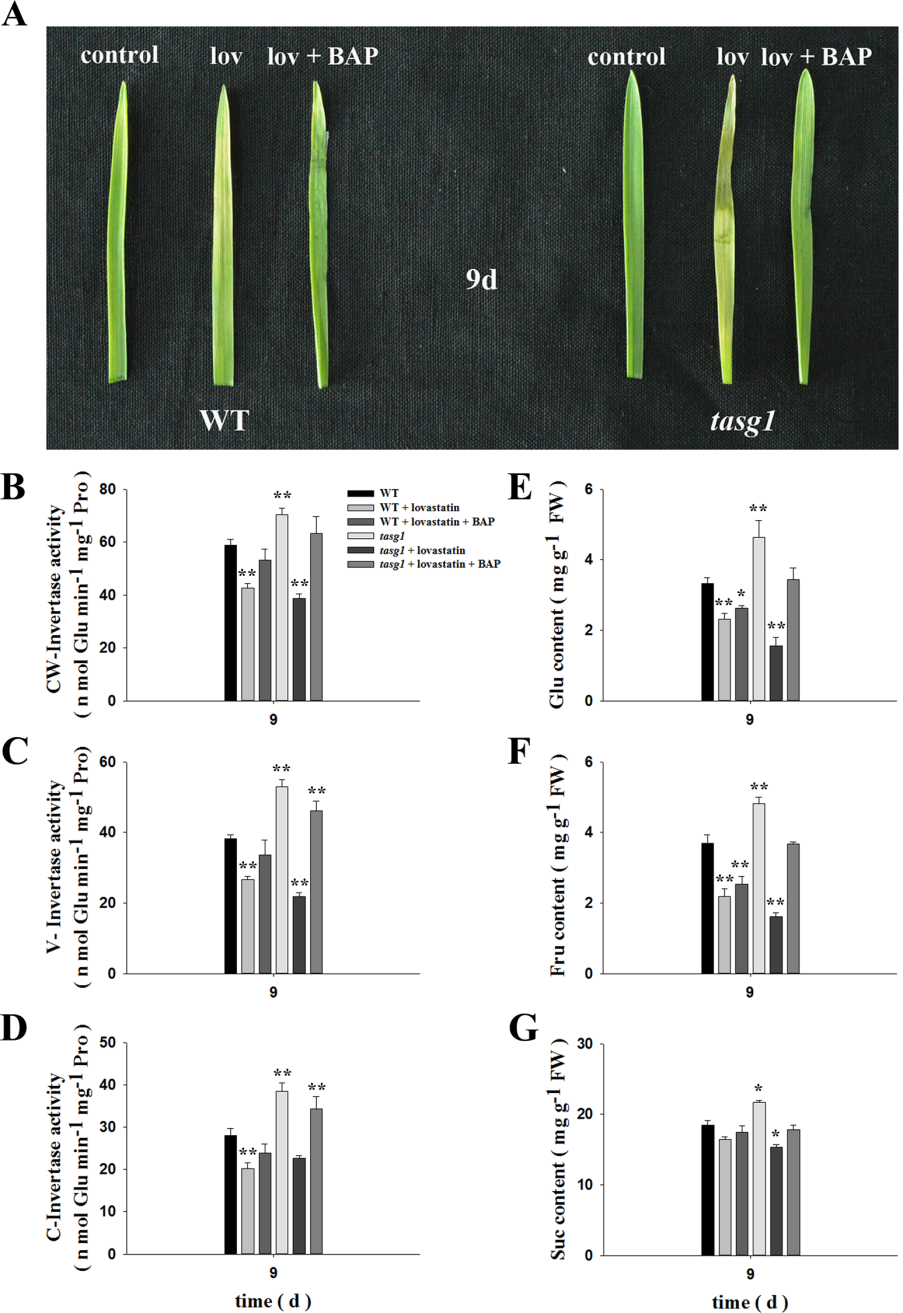
Effects of lovastatin on invertase activity in WT and *tasg1*. Seedlings of WT and *tasg1* were incubated in water without nutrients. The samples were from the second leaf of WT and *tasg1* at 9 d of growth. Changes in the (A) leaf phenotype, (B) CW-Invertase, (C) V-Invertase, (D) C-Invertase. Lovastatin concentration used was 40 μM L^-1^. BAP concentration was 100 μM L^-1^. Error bars indicate ± SE of the mean of data from three replicates. *, *P* < 0.05; **, *P* < 0.01.

We tested the involvement of CK in regulating the activity of invertase and the content of soluble sugar in *tasg1* (Fig 10). Under normal conditions, in the absence of lovastatin, the leaves of *tasg1* displayed obvious stay-green phenotype. However, when CKs were inhibited with lovastatin, premature senescence phenotype appeared in *tasg1* unlike in WT; on treating with 6-benzylaminopurine (BAP), partial recovery from the premature senescence phenotype was observed in *tasg1* (Fig 10A). Moreover, under normal conditions in the absence of lovastatin, the activities of CW-Invertase (Fig 10B), V-Invertase (Fig 10C), and C-Invertase (Fig 10D) in *tasg1* were significantly higher (by 20.1, 38.6, and 37.1%, respectively) than those in WT. The trend of accumulation of Glu (Fig 10E), Fru (Fig 10F), and Suc (Fig 10G) was basically the same as that of the increase in invertase activity; their content were 39.4, 30.3, and 17.6% higher, respectively, in *tasg1* compared to that in WT, respectively. When treated with lovastatin, the activities of CW-Invertase, V-Invertase, and C-Invertase were decreased and the decrease in *tasg1* was higher than that in WT. However, the activities of these enzymes were significantly increased in *tasg1* upon treatment with BAP. This trend was consistent with the changes in the Glu, Fru, and Suc content. These results suggest that the high invertase activity in *tasg1* might be regulated by the high CK levels (Fig 3).

## Discussion

For centuries, selective breeding has played an important role in increasing the crop yield and improving the product quality [29]. The yield is often reduced because of less photosynthesis when premature senescence occurs, but it is also lost if the resources are not efficiently recycled to the grain at the end of the growing season. The latter cause is related to the metabolism and transportation of sugars.

In a previous report, we demonstrated that *tasg1* could retain high photosynthetic capacity and inhibit chlorophyll degradation at the late developmental stage [19]. Furthermore, CK metabolism was altered in *tasg1* and was involved in their stay-green phenotype [20]. In the present study, we observed that the biomass accumulation in *tasg1* was higher than that in WT, as indicated by their larger stem and higher leaf weight (S1 Fig). Photosynthesis is a key underlying factor regulating plant biomass. At later filling stages, especially from 21 to 32 d after flowering, Pn was always higher in *tasg1* than in WT (Fig 2A), coinciding with higher Gs, E, Fv/Fm, and ΦPSII values (Fig 2C–2F), which suggested a greater photosynthetic capacity in *tasg1* than in WT at the filling stage. The greater photosynthetic capacity might play a major role in *tasg1* having higher biomass than WT (S1 Fig).

### CK-regulated cell division might be involved in the larger volume of grains at the early filling stage in *tasg1*

The role of the CK is important in the regulation of different cell cycle phases [8]. The inhibition of bud outgrowth in the tiller inhibition (*tin*) mutant is associated with a reduction in the expression of marker genes, *HIS4* and *PCNA* for S, and *CDKB* for M phases of the cell cycle [27]. In our previous study, we described the association of the stay-green phenotype of *tasg1* with altered CK metabolism [20]. In the present study, we found that the levels of active CK were higher in the grains of *tasg1* at the early filling stage (7 d) than those of WT (Fig 3D–3F). The expression of *CDKB* (Fig 4A) and *CycD2* (Fig 4D) might play an important role in the enhancement of the grain volume (sink) in *tasg1*. We, therefore, speculated that the increased content of active CKs could be involved in the larger grain volume of *tasg1* by regulation of mitosis (Fig 4) at the early filling stage (Fig 1B).

### Changes in carbohydrate metabolism could be an important component in the stay-green phenotype and the smaller grain weight of *tasg1*

Carbohydrates produced by photosynthetically active source leaves are transported to heterotrophic sink tissues of the plant, such as those present in the growth zones and storage organs. Partitioning of photoassimilates from source organs to various sinks is under strict developmental control [30]. Maria et al. [31] reported that the stay-green phenotype was related to Suc metabolism and transportation in transgenic tobacco (*Nicotiana tabacum*) plants.

We observed that the concentrations of soluble sugars in *tasg1* (flag leaf and grain) at the late filling stage were significantly higher than those in WT (Fig 5). The content of Suc in the flag leaf of *tasg1* was consistent with the photosynthetic capacity (Fig 2). The high content of Suc in *tasg1* at the late filling stage was consistent with the up-regulated expression of a Suc-inducible gene and downregulation of a marker gene for Suc starvation (Fig 6). The higher content of soluble sugars in *tasg1* (Fig 5) implied that more soluble sugars were transported to sink tissues in *tasg1*. On the contrary, the grains of *tasg1* were smaller than those of WT (Fig 1B), suggesting a low conversion rate of soluble sugars to starch in *tasg1* at the late filling stage. This indicates that more soluble sugars were retained in the vegetative organs of *tasg1*, such as leaves and stems. Indeed, Fru pools in the vegetative tissues preserve the carbon flux in the kernel when the transport of the synthesized photosynthesis products is insufficient [32]. The increased content of Fru in *tasg1* might be one of the reasons for larger biomass in the late growth stage. Sufficient nutrients in the vegetative organs of *tasg1* could be one of the most important components responsible for the stay-green characteristic of *tasg1*.

Suc turnover is accomplished by the activity of the Suc-metabolizing enzymes, such as SPS and SuSy. Suc is predominantly synthesized by SPS, and is subsequently cleaved, mainly by SuSy [33]. The higher activities of SPS and SuSy in *tasg1* also suggest an active carbohydrate metabolism in the leaves and grains at the late filling stage (Fig 7).

### CK-regulated invertase activity might be one of the most important factors involved in the stay-green phenotype of *tasg1*

The activity of CW-Invertases at the site of phloem unloading has been proposed to be a major factor controlling the sink strength by drawing Suc [14]. Our data for the flag leaves show that the activities of all the three types of invertases were higher in *tasg1* than in WT at the late filling stage (Fig 8A–8C). However, in the grains, only CW-Invertase activity was higher in *tasg1* (Fig 8D), suggesting that Suc was actively utilized in *tasg1*. Surprisingly, the study showed that the grains were smaller in *tasg1* at the late filling stage (Fig 1B). We propose that the ineffective conversion from soluble sugars (Glu) to starch might be involved in the smaller grain size.

The expression of *Ivr1* and *Ivr3*, which code for CW-Invertase, was significantly elevated in *tasg1* in different tissues at the late filling stage (Fig 9). These data suggest that, in addition to the leaves and grains, carbohydrate metabolism was also active in other organs in *tasg1*. When excess Suc is decomposed into Glu and Fru and is retained in different tissues instead of grains at the late filling stage, the senescence of vegetative organs is delayed [30]. The higher biomass of *tasg1* than that of WT also supports this notion (S1 Fig).

In a previous study, CKs were shown to upregulate the expression of genes encoding cell wall invertases [34], and the activity of invertase was essential for CK-induced nutrient redistribution and delayed senescence [35]. Herein, we studied the involvement of CKs in the invertase activity in *tasg1* seedlings. When treated with the CK inhibitor lovastatin, both WT and *tasg1* seedlings exhibited premature senescence phenotype and the activity of invertase was significantly decreased (Fig 10). However, the effects of lovastatin were greater on tasg1 than on WT, suggesting a major function of CKs in the activity of invertase and stay-green phenotype of *tasg1*. When treated with BAP, the premature phenotype was partially recovered and the activities of CW-Invertase, V-Invertase, and C-Invertase were increased in *tasg1*. Concomitantly, the contents of Glu and Fru were altered in WT and *tasg1*; however, the change was more prominent in *tasg1*. These results reinforce the opinion that extracellular invertase and soluble sugars (Glu and Fru) are essential components of cytokinin-mediated delay in senescence [31] and also support the view that invertases provide an important link between phytohormone action and soluble sugar metabolism [17].

## Conclusion

**Fig 11:**
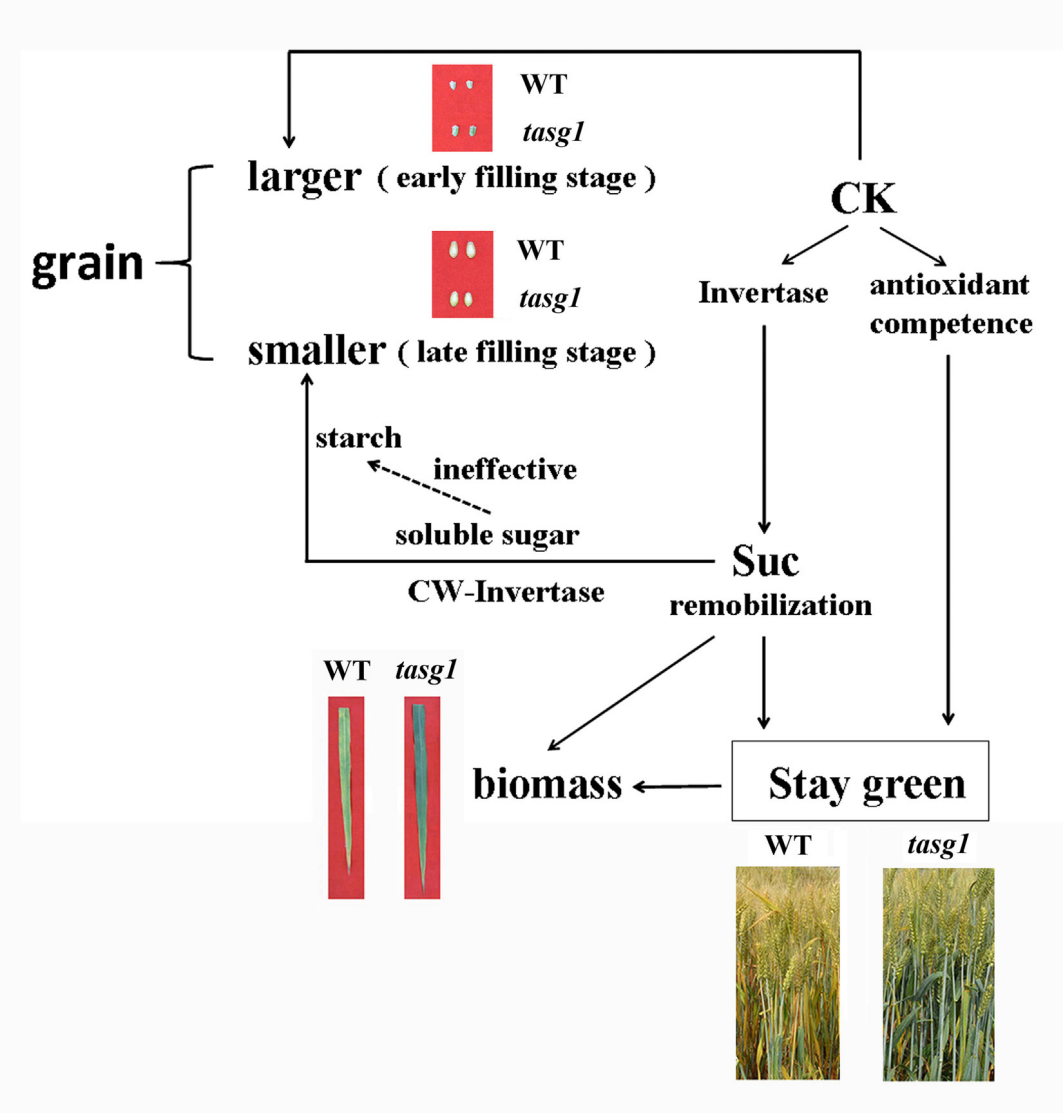
Proposed model for stay-green phenotype in *tasg1*.

Based on the results of the present study, as well as those reported by us earlier [20], wherein we related the CK-regulated antioxidant competence to the stay-green phenotype of *tasg1*, we hereby propose a model that might be involved in the stay-green phenotype of *tasg1* (Fig 11). Suc content in the leaves of *tasg1* was higher than that in WT because of its greater capacity for active photosynthesis. However, excess Suc was decomposed into Glu and Fru by invertase, following which the soluble carbohydrate was retained in different tissues, rather than in the grains, at the late filling stage. This resulted in the stay-green phenotype and higher biomass of *tasg1* but was responsible for smaller grain volume in this mutant. The invertase activity regulated by CKs was directly or indirectly involved in Suc remobilization and the stay-green phenotype of *tasg1*. The ineffective conversion from soluble sugars to starch might be involved in the smaller grain size in *tasg1*.

## Supporting Information

S1 Fig The dynamics of single stem biomass between *tasg1* and WT during the filling stage in the field. (A) Stem weight, (B) Leaf weight. Error bars indicate means ± SE of data from thirty replicates. *, *P* < 0.05; **, *P* < 0.01.
(TIF)
