# Supplementary figures and images for: Cytokinin-Regulated Sucrose Metabolism in Stay-Green Wheat Phenotype
Source: PLoS One. 2016 Aug 31;11(8):e0161351. doi: 10.1371/journal.pone.0161351 (PMC5007033; doi:10.1371/journal.pone.0161351)

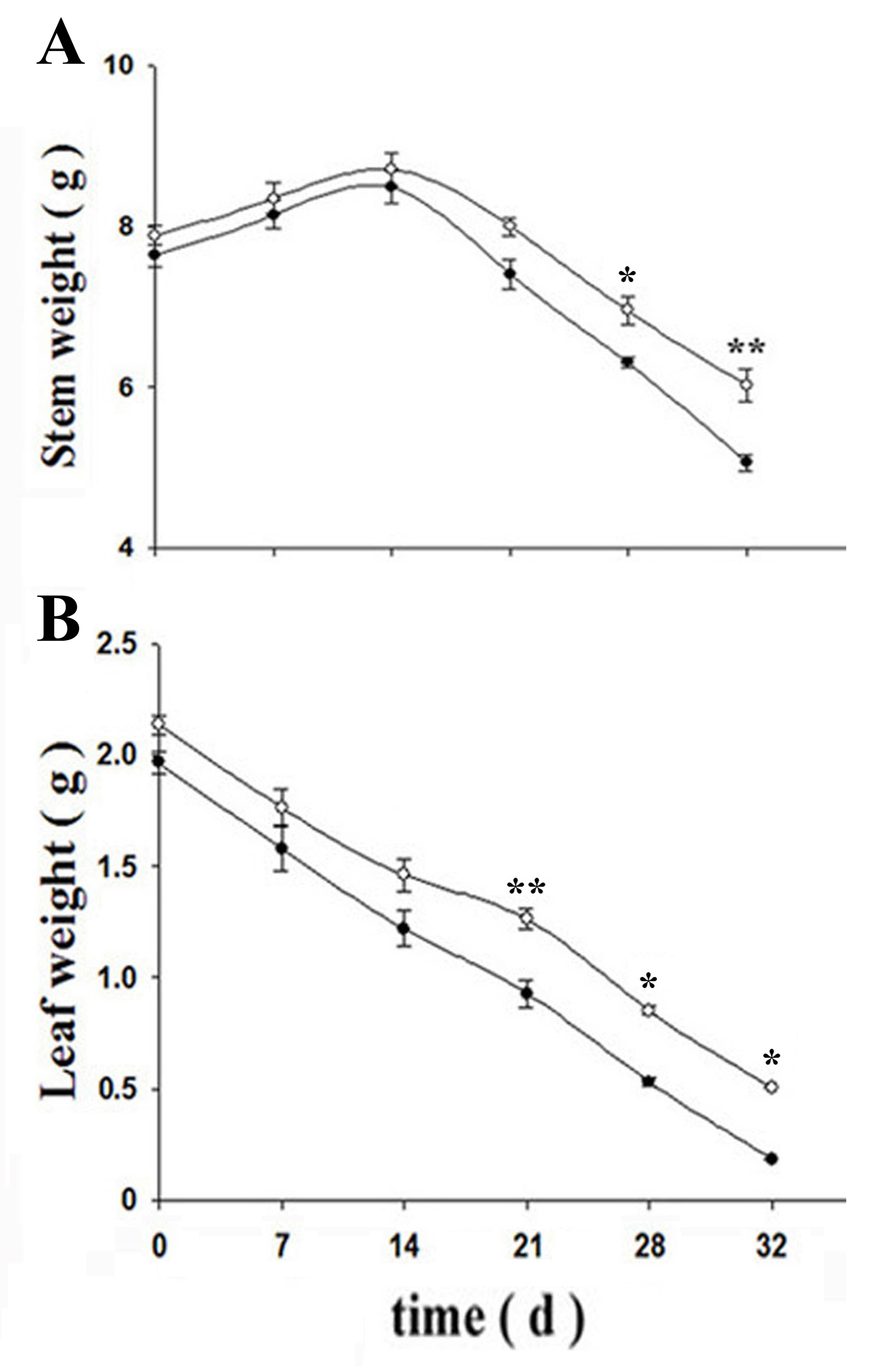

Supplement: S1 Fig — (A) Stem weight, (B) Leaf weight. Error bars indicate means ± SE of data from thirty replicates. *, P < 0.05; **, P < 0.01. (TIF) [file pone.0161351.s001.tif]
